# Essential oil from *Sabina chinensis* leaves: A promising green control agent against *Fusarium* sp.

**DOI:** 10.3389/fpls.2022.1006303

**Published:** 2022-11-11

**Authors:** Jianyun Zhang, Ziyi Zhao, Wenyu Liang, Jingyi Bi, Yuguang Zheng, Xian Gu, Huiyong Fang

**Affiliations:** ^1^ College of Pharmacy, Hebei University of Chinese Medicine, Shijiazhuang, China; ^2^ Traditional Chinese Medicine Processing Technology Innovation Center of Hebei Province, Hebei University of Chinese Medicine, Shijiazhuang, China; ^3^ International Joint Research Center on Resource Utilization and Quality Evaluation of Traditional Chinese Medicine of Hebei Province, Shijiazhuang, China; ^4^ Department of Resources Science of Traditional Chinese Medicines, School of Traditional Chinese Pharmacy, China Pharmaceutical University, Nanjing, China; ^5^ Department of Pharmaceutical Engineering, Hebei Chemical and Pharmaceutical College, Shijiazhuang, China

**Keywords:** *Sabina chinensis*, essential oil, response surface methodology, gas chromatography-mass spectrometry, antifungal activity, soil-borne disease

## Abstract

*Sabina chinensis* is a woody plant with important ecological functions in different regions of China, but its essential oils (EO) against plant pathogenic fungi remain largely undetermined. The purpose of our study was to assess the chemical composition and antifungal activity of *S*. *chinensis* EO based on optimization of the extraction process. In this study, an actionable and effective model with the experimental results and identified optimum conditions (crushing degree of 20 mesh, liquid–solid ratio of 10.1:1, immersion time of 9.1 h) was established successfully to achieve an extraction yield of 0.54%, which was basically consistent with the theoretical value. A total of 26 compounds were identified using headspace gas chromatography–mass spectrometry (GC–MS) and showed that the major constituent was *β*-phellandrene (26.64-39.26%), followed by terpinen-4-ol (6.53-11.89%), bornyl acetate (6.13-10.53%), etc. For Petri plate assays, our experiments found for the first time that *S. chinensis* EO revealed high and long-term antifungal activity against the tested strains, including *Fusarium oxysporum* and *Fusarium incarnatum*, at EC_50_ values of 1.42 and 1.15 µL/mL, which especially reached approximately 76% and 90% growth inhibition at a dose of 0.2 µL/mL, respectively. Furthermore, the antifungal activity of EO from different harvest periods showed remarkable variation. The orthogonal partial least-squares discriminant analysis (OPLS-DA) method revealed 11 metabolites with chemical marker components, and 5 of its potential antifungal activities, terpinen-4-ol, *α*-terpineol, *α*-elemol, *γ*-eudesmol, and bornyl acetate, were strongly correlated with the mycelial inhibition rate. In total, this study explored the antifungal activity of EO against root rot fungus as a potential fungicide and provided valuable information into developing potential products from natural agents.

## Introduction

Soil-borne plant diseases are increasingly causing a considerable destructive impact on agricultural production systems worldwide and increasing the incidence of soil disease with a direct huge economic loss for farmers (Wei et al., 2015; Delgado-Baquerizo et al., 2020; Yuan et al., 2020). Among these, root rot caused by fungal pathogens such as *Fusarium oxysporum* and *Fusarium incarnatum* has continued to be a primary production and quality limitation in global crop production and even the culture of herb materials in China (Chand et al., 2016; Deng et al., 2022). At present, the main method for controlling medicinal herbs and crop diseases remains highly dependent on chemical fungicides (Liu et al., 2017). However, these extensive applications of chemical fungicides cause serious pollution problems and threaten human health owing to increased inevitable exposure to fungicides from field, food, and water sources. Resistance to insecticides could result in several billion dollars in the agricultural sector annually worldwide and even limited effects due to evolving resistance (Gould et al., 2018). Moreover, biocide poisoning not only increases the risk of numerous health disorders, such as endocrine disruption, Alzheimer’s disease and cancers, in humans but also directly causes nearly 300,000 deaths worldwide (Freire et al., 2013; Yan et al., 2016; Sabarwal et al., 2018). Thus, it is urgent to find a more efficient and environmentally compatible alternative to control pathogenic microorganisms in agriculture.

In recent years, essential oils (EO) from plants against pathogenic fungi have received increased attention owing to their natural active ingredients, which have the advantages of easy degradation, low environmental pollution, high efficiency, and easy development (Dorman and Deans, 2000; Pateiro et al., 2021). Importantly, EO contain complicated components and show multiple target synergistic effects to exert antipathogen effects, so it is not easy to develop drug resistance (Hou et al., 2022). Furthermore, some constituents occurring in the EO, including carvacrol and thymol (Zhao et al., 2021), viridiflorol, pinocarveol, bornyl acetate, ledol (El Karkouri et al., 2021), citral and geraniol (López-Meneses et al., 2017), have been found to have antifungal activity. It has been previously reported that many species are important EO sources and have potent antimicrobial effects, such as *Allium sativum*, *Foeniculum vulgare* (Li et al., 2022a), *Eucalyptus camaldulensis* (Gakuubi et al., 2017), and *Artemisia annua* (Ma et al., 2019), but *Sabina chinensis* is relatively unknown. Of traditional Chinese plants, the *S*. *chinensis* tree is widely distributed throughout the northern region of China for air quality improvement on roadsides and parks, and its pollen also has strong allergenicity to the pollen allergic population (Cui et al., 2022). Unfortunately, the leaves of *S*. *chinensis* have not been fully utilized and developed, which wastes large biomass resources. Other previous studies have only focused on EO extracted from *Cupressus sempervirens* L., *Juniperus chinensis*, and *Juniperus seravschanica*, which exhibit high antibacterial activities, whereas research on inhibiting numerous root rot diseases has not been previously reported (Rguez et al., 2019; Khamis and Chai, 2021). As such, *S*. *chinensis* leaves may be a chance to search for effective alternatives in disease control and reduce future fungicide costs.

Consider the case of classical methods for hydrodistillation with simplicity in installation and ease of implementation, which are widely used in terms of experimental research and industrial scale (Wu et al., 2019). In the Chinese pharmacopoeia, the hydrodistillation process is also a recommended evaluation method to determine the EO content of drugs (e.g., *Angelicae sinensis* Radix., *Forsythia suspensa*, and *Schizonepeta tenuifolia*) (Commission, 2020). However, this simple heating process makes it difficult to adequately extract EO due to the complexity of plant tissues. Response surface methodology (RSM) based on combining experimental design with mathematical modelling has been widely used to process optimization methods (Bas and Boyacı, 2007). It has also been made to improve the extraction efficiency of EO, for instance, in *Origanum vulgare* L. and *Citrus latifolia* (Tan et al., 2012; Wu et al., 2019). For this purpose, this study will establish optimal extraction conditions using hydrodistillation combined with regression equations to evaluate the extraction efficiency of *S*. *chinensis* EO.

Hence, the objective of this study was to explore the optimal extraction process using the RSM method and to identify whether EO from *S. chinensis* leaves possesses antifungal properties. The *in vitro* antifungal activity of EO from different harvest periods was compared to further analyze the possible active ingredient and antimicrobial properties using gas chromatography–mass spectrometry (GC–MS). The results identify compounds in the *S*. *chinensis* EO that have the main antifungal activity, which could potentially be used against the root rot disease of plants. This study provides new insight into the rational development of plant fungicides simultaneously.

## Materials and methods

### Sample materials and fungal pathogens

Plants of *Sabina chinensis* (Linn.) Ant. var. chinensis (*S*. *chinensis*) were collected from trees grown in the herbal garden of Hebei University of Chinese Medicine in Hebei Province, China (115°19′58.05E, 38°24′27.64N). To ensure the standardization of all sample-collection conditions, fresh leaf samples were collected from mature, healthy plants, approximately 20 cm in diameter, from the bottom and middle with scissors. The leaves of *S*. *chinensis* are scaly when mature and are manually discarded in the process of growth (Kang et al., 2019). The botanical identification of the plant was performed by a botanist, Professor Yun-Sheng Zhao. A voucher specimen of the plant was deposited at our herbarium of Hebei University of Chinese Medicine under the number ZYF022364. All fresh leaf samples of *S*. *chinensis* were harvested in March, April, and May 2022 to investigate the influence of different harvest periods on the chemical composition and antifungal activity. The fresh leaves were chopped into 0.5-1 cm segments and dried within the shade under natural conditions to a water content of approximately 5-10%. Then, the dried leaf samples were packaged, labeled, and stored in the herbarium during the experiment. These fungal strains of *Fusarium incarnatum* and *Fusarium oxysporum* were obtained from Dr. Bo Zhu, Zhejiang Chinese Medicine University, Zhejiang Province, China (Wu et al., 2021). Fungal pathogens were maintained on potato dextrose agar (PDA) slants at 4°C.

### Extraction and optimization of process parameters

EO was extracted from *S*. *chinensis* leaves by hydrodistillation, for which leaf samples were placed in a flask with a capacity of 1000 mL and extracted with reverse-osmosis (RO) water. The EO was then collected, dried with anhydrous sodium sulfate and stored at 4°C. On the basis of the preexperiment, taking the yield of EO from *S. chinensis* leaves as the evaluation index, a single-factor experiment was adopted to investigate the crushing degree (16, 20, 24, 30, 40), liquid–solid ratio (8:1, 10:1, 12:1, 14:1, 16:1) and immersion time (3, 6, 9, 12, 15). A Box–Behnken scheme was established by Box–Behnken Design Expert V8.0.6 software (State-Ease Inc., USA), in which the parameters under consideration varied among three levels: low, middle, or high based on single-factor experimental data (**Supplementary Table 1**). All experiments were performed three times. The statistical significance and regression coefficients were evaluated to test the validity of the model and estimated parameters (Tan et al., 2012).

The extraction yield of EO was calculated based on the dried weight of the leaves using the following formula (1):


(1) $$YEO=\;\frac{WEO}{WDL}\times 100$$

where YEO is the yield of essential oils (%), WEO is the weight of the essential oils (g), and WDL is the weight of dry leaves (g).

### Headspace gas chromatography–mass spectrometry and component alignment

The EO was analyzed by GC–MS using an Agilent 7890B-5977B system equipped with a headspace automatic sampler and FID detector (Agilent Technologies Inc., USA) (Yuan et al., 2021). GC separations were carried out on an Agilent HP-5 MS capillary column (30 m × 0.25 mm, 0.25 μm) with high purity hydrogen as the carrier gas (1.0 mL/min) at a splitting ratio of 1:20. The oven temperature program was as follows: 52°C held for 1 min, followed by temperature increases from 52°C to 100°C at 4°C/min and then held for 1 min. Then, it was increased from 100°C to 140°C by 10°C/min and kept for 2 min, increased from 140°C to 150°C by 2°C/min and kept for 1 min. Finally, it was increased to 200°C by 25°C/min and kept for 1 min. The detailed mass spectrometry conditions were as follows: EI source operated at 70 eV; acquisition mass range from 50 to 500 m/z; ion source temperature at 230°C; quadrupole temperature at 150°C; solvent delay of 5 min.

Each EO sample was prepared by diluting 10 times with pure N-hexane chromatography (99.99%, Aladdin, China). After drying anhydrous sodium sulfate (≥99.0%, Tianjian Yongda Chemical Co., Ltd), a precision suction of 50 μL EO was placed in 20 mL Agilent headspace glass vials. Compared to the total peak area and GC retention times, the peak area percentage of the EO constituents was detected using MassHunter Quantitative Analysis software (version B.09, Agilent). The main chemical compounds of EO were tentatively identified based on the comparison of their GC Kovats retention indices (RIs) with reference to a homologous series of C8-C20 n-alkanes (Cazella et al., 2019). The mass spectra of each compound were matched with authentic standards from the NIST17 library data and with those reported in the literature (Wu et al., 2019).

### Antifungal activity by airtight fumigation assay

In this study, the antifungal activity of EO at different concentrations was assessed using an airtight fumigation assay with modifications (Li et al., 2020). Sterilized Petri dishes (90 mm diameter) were filled with 10 mL of PDA (potato dextrose agar) medium for preparation. Then, a 5 mm diameter pathogenic fungi cake was obtained with a hole punch at the edge of the pathogen colony and inoculated in the center. Sterile filter paper (20 mm diameter) with tweezers was placed in the center of the dish cover, and the doses of 2.5, 5, 10, 15, and 20 μL EO were removed with a pipette gun. The residual volume of each Petri dish was calculated as approximately 100 mL, and the final concentration in each dish reached 0.025, 0.05, 0.10, 0.15, and 0.20 µL/mL to evaluate the antifungal profiles. Petri dishes were inverted, sealed with parafilm, and incubated in an incubator (Yiheng Instrument Co., Ltd., Shanghai, China) at a constant temperature of 28°C for 120 h. The inhibitory antifungal activity in each treatment was recorded at 24 h intervals and measured using the mycelial growth rate method (Chutia et al., 2009) until the end of the experiment. For each treatment, three repeats were carried out. The antifungal activity of *S. chinensis* EO from different harvest periods was compared according to the above method. The doses of 5 μL EO from *S*. *chinensis* in March, April, and May were added to the filter paper, and the assay plates were incubated at 28°C for 120 h. The rest was the same as above.

### Statistical analysis

All data are presented as the mean values and standard deviation (SD). Data were checked for normality using the Shapiro–Wilk method and by visualizing the histogram. Data on the inhibitory effect of EO on mycelial growth were subjected to analysis by one-way analysis of variance (ANOVA) using SPSS 26.0 (SPSS Inc., USA) (Moghaddam et al., 2015). Mean comparisons were made using Duncan’s multiple range tests at *P<* 0.05. The EC_50_ values with their confidence intervals were determined with GraphPad Prism 8.0.2 software. Data preprocessing is imperative to ensure that the relative content of compounds in the GC–MS data is standardized and entirely comparable using Agilent MassHunter Quantitative Analysis B.09.00 software (Li et al., 2022b). An orthogonal partial least square discriminant analysis (OPLS-DA) model was established with chemical components as variables using SIMCA-P software (version 14, Umetrics). The explanatory power of the models was evaluated by calculating the cumulative modeled variation in the marginal *R* ^2^ (cum) and the predictive ability parameter *Q* ^2^ (cum) (Su et al., 2021). For correlation analysis, Spearman’s correlation coefficient was calculated and analyzed with Origin 2019 software (Origin Lab, Northampton, MA, USA).

## Results and discussion

### BoxBehnken design analysis

EO plays an important role in the pharmacological, food processing, biopesticide, and industrial fields because of its complicated and varied chemical composition and extensive pharmacological activities. Previous research showed that the extraction method not only affects the content and composition of EO but is also critical in enhancing process yields, understanding biological activities, and further applications, especially for the components accounted for less (Taticchi et al., 2021). Therefore, the selection and optimization of extraction parameters were first performed.

The response surface method (RSM) is a statistical comprehensive testing technique that uses deterministic experiments and multivariate quadratic regression equations to fit, which can reduce the number of tests and solve the multivariable factors (Ferreira et al., 2007). Therefore, the Box–Behnken model was used to design the experiment based on the results of the single-factor experiment. Finally, crushing degree (16, 20, 24), liquid–solid ratio (8:1, 10:11, 12:1), and immersion time (6, 9, 12) are selected as the best horizontal range. Using the Box–Behnken experimental design, 17 experimental groups of three factors and a three-level optimization model were obtained. The Box–Behnken design of each factor and level is shown in **Supplementary Table 1**. Our mathematical model can be expressed as the following model (2):


(2) $$Y=0.54\;-\;0.029A+\;0.005B+\;0.002C-\;0.019AB-\;0.005AC-\;0.016BC-\;0.058A^{2}-0.078B^{2}-\;0.037C^{2}$$

**Figure 1:**
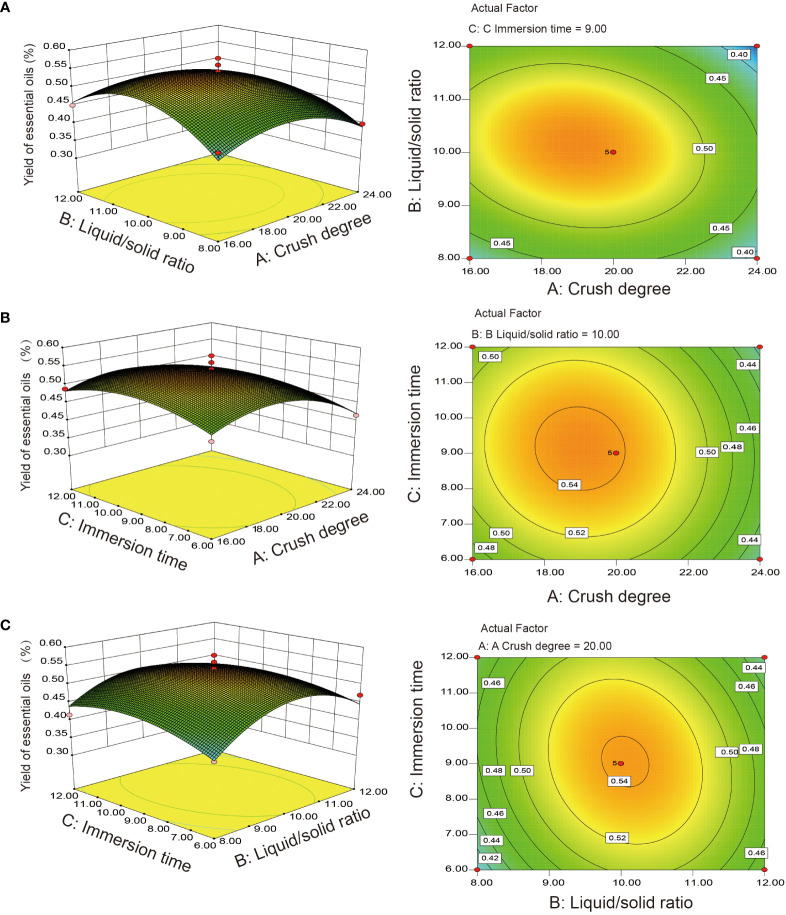
Response surface plots and contour map for the interaction analyzes. **(A)** Crush degree and liquid–solid ratio; **(B)** Crush degree and immersion time; **(C)** Liquid–solid ratio and immersion time.

The statistical significance and regression coefficients were evaluated based on ANOVA with the *F* test and *P* value (**Table 1**). The regression equation model showed that the model was significant (*P* = 0.0061< 0.01). The lack-of-fit statistic was not significant (*P* = 0.4455 > 0.05), indicating that the model can be matched with the experimental data. The coefficient of determination (*R* ^2^ = 0.9110) revealed good fitness and reliability for matching the model equations (*Y*) to actual measurements (yield). The *p* value of each model term indicated that factor (*A*) and interactive coefficients (*A* ^2^, *B* ^2^, *C* ^2^) were significant and constituted the model between the interactive term and independent variables. Contour plots and response surfaces of the effect of three factors and three levels on the extraction yield of EO are shown in **Figure 1**. We can conclude that the crush degree had the largest effect on the yield of EO, followed by the liquid–solid ratio and immersion time (**Figure 1** and **Table 1**). According to the mathematical model, the optimal extraction conditions of RSM were predicted as follows: crushing degree 18.95 mesh, liquid–solid ratio of 10.1:1, immersion time 9.10 h, theoretical yield 0.55%. To validate the model accuracy and feasibility, triplicate extraction experiments of process conditions were performed as follows: crushing degree 20 mesh, liquid–solid ratio of 10.1:1, immersion time 9.1 h. The actual yield of EO was 0.54%, which was basically consistent with the theoretical value, and the error was small. Overall, the design model could be effectively used to optimize the extraction efficiency for the extraction yield of *S*. *chinensis* EO.

**Table 1:** Variance analysis results of the regression equation.

Source	Sum of squares	df	Mean square	*F* value	*P* value
Model	0.061	9	6.730×10^-3^	7.96	0.0061
*A*	6.844×10^-3^	1	6.844×10^-3^	8.09	0.0249
*B*	2.000×10^-4^	1	2.000×10^-4^	0.24	0.6416
*C*	3.200×10^-5^	1	3.200×10^-5^	0.038	0.8513
*AB*	1.406×10^-3^	1	1.406×10^-3^	1.66	0.2382
*AC*	1.102×10^-4^	1	1.102×10^-4^	0.13	0.7287
*BC*	1.056×10^-3^	1	1.056×10^-3^	1.25	0.3006
*A2*	0.014	1	0.014	16.97	0.0045
*B2*	0.026	1	0.026	30.59	0.0009
*C2*	5.882×10^-3^	1	5.882×10^-3^	6.96	0.0336
Residual	5.919×10^-3^	7	8.456×10^-4^		
Lack of fit	2.679×10^-3^	3	8.930×10^-4^	1.10	0.4455
Pure error	3.240×10^-3^	4	8.100×10^-4^		
Total	0.066	16			
R^2^	0.9110				
Adj-R^2^	0.7965				
C.V. %	6.32				
Ade precision	8.060				

A is the crush degree (mesh); B is the liquid–solid ratio (mL/g). C is the immersion time (h).

Our results showed that crushing degree was a major factor in the optimal extraction parameters of *S*. *chinensis* EO, consistent with a previous study (Gong et al., 2009). Dissolution, diffusion, and infiltration are believed to be essential for improving the extraction yield related to EO from broken plant tissue structures in hydrodistillation extraction processes (Díaz-Suárez et al., 2021). The EO yield extracted from *S*. *chinensis* leaves by optimizing the extraction parameters reached 0.54%, which was higher than that obtained by Gong et al. (2009) and slightly lower than Zhao obtained by the microwave-assisted extraction method (Zhao, 2018). This could be due to the difference in the investigated factors, such as enzymolysis temperature and time, and ultrasound-assisted extraction also affects the EO yield owing to mechanical and thermal effects that cause the rupture of plant cell walls (Vila Verde et al., 2018).

### Antifungal activity of *Sabina chinensis* essential oil

**Figure 2:**
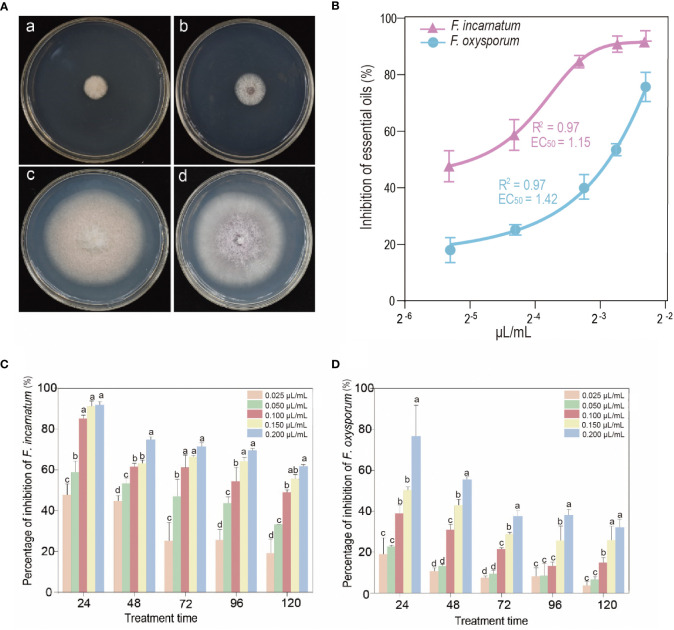
**(A)** Antifungal activity of the EO of *Sabina chinensis* leaves tested by the airtight fumigation method against *Fusarium incarnatum* **(a)** and *Fusarium oxysporum* **(b)**; **(c, d)** are controls for *F. incarnatum* and *F. oxysporum*, respectively. **(B)** Inhibitory rate after an airtight fumigation assay test with different doses of *S. chinensis* EO. The inhibitory effect of EO (means ± SDs) against *F. incarnatum* (pink curve) and *F. oxysporum* (blue curve). **(C, D)** The percentage of inhibition with different treatment times and doses of EO. Vertical bars represent the standard errors. Different lowercase letters indicate that there are significant differences between different treatments at the same time (*P*< 0.05).

The properties against the root rot fungus strains, including *F. oxysporum* and *F. incarnatum*, of EO after inoculation with different doses and treatments are shown in **Figure 2**. The experimental results revealed that *S*. *chinensis* EO has a high antifungal potential against pathogenic fungi using only a low concentration to the effective concentration of inhibiting 50% of mycelial growth (EC_50_ values). The EO showed high efficiency against *F*. *oxysporum* with EC_50_ values of 1.42 µL/mL. Furthermore, the EO also showed higher antifungal activity against *F*. *incarnatum*, with an EC_50_ value of 1.15 µL/mL. Our study reported is consistent with previous findings, which tested the EO of *Thymus vulgaris* grown in Iran for *in vitro* antifungal activity against *F. oxysporum* and *Drechslera spicifera* (Moghaddam and Mehdizadeh, 2020). Additionally, EO inhibited mycelial growth in all tested strains selected and correlated with the effective time and dose-dependent manner. In Petri plate assays, at a higher concentration with EO at 0.2 µL/mL within 24 h, the inhibition of mycelial growth against *F*. *incarnatum* reached approximately 90%, and *F*. *oxysporum* reached 76%. The antifungal properties are more effective against *F*. *incarnatum* than *F*. *oxysporum*. It is worth noting that the EO concentration (from 0.1 to 0.2 µL/mL) within 120 h inhibited the mycelial growth of *F*. *incarnatum* by approximately 48.76% to 60.29%, but *F*. *oxysporum* reached 14.61% to 32.03%. Although the trend of antifungal activity declined with increasing treatment time, it still showed inhibitory effects. The results in this study are also similar to those of the study obtained by Parikh et al. (2021), who determined that the seven essential oils inhibited mycelial growth of all tested pathogens, including *Fusarium avenaceum*, by 50 to 100%. López-Anchondo et al. (2021) also reported that the EO from *Prosopis glandulosa* controls the growth rate reduction of *Colletotrichum gloeosporoides* (74.92%) and *F*. *oxysporum* (64.82%) but was less efficient against *in vivo* conditions. Another recent study revealed that Cumin EO induced the expression of most genes against *F. oxysporum* from *Panax notoginseng* pathogens (Huo et al., 2021), which provided a theoretical basis for controlling root diseases at the transcriptional level.

### GC–MS analysis of *Sabina chinensi*s essential oil components

**Figure 3:**
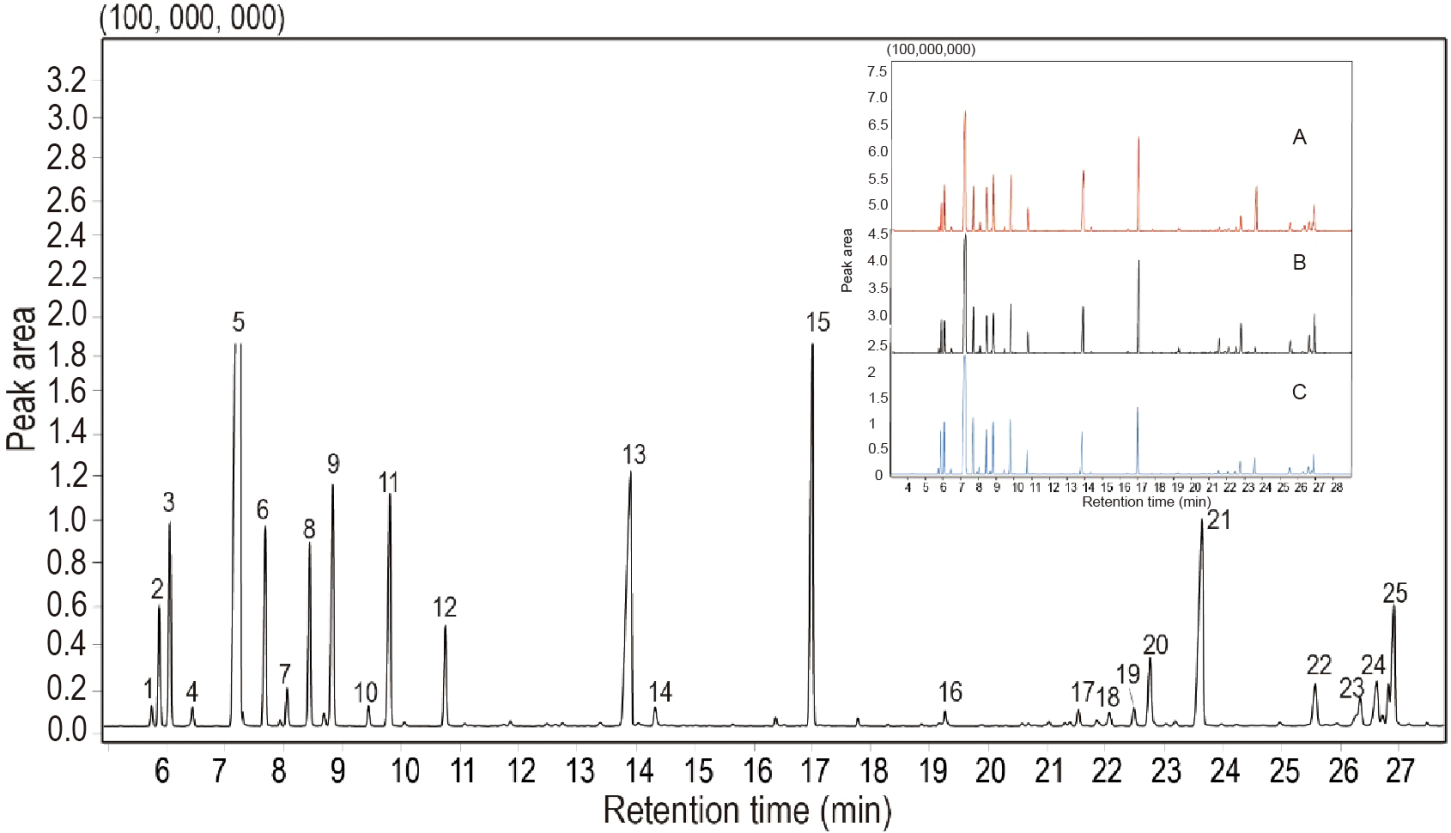
Total ion current chromatogram of essential oils from *Sabina chinensis* leaves analyzed using a gas chromatograph-mass spectrometer. Zoomed areas view insets show the chemical composition of TIC identified from March **(A)**, April **(B)**, and May **(C)** samples. Peak numbers (No.) correspond to the main compounds mentioned in **Table 2**.

In the present study, 26 compounds were identified in the EO from *S*. *chinensis* leaves by GC–MS analysis (**Table 2** and **Figure 3**). The major constituent and the relative content of the EO were *β*-phellandrene (26.64-39.26%), followed by terpinen-4-ol (6.53-11.89%), bornyl acetate (6.13-10.53%), *α*-terpinene (3.35-4.53%), and *β*-pinene (4.20-5.69%). Compared with previous studies, the chemical composition of EO was close, but the percentages of those constituents were different (Gong et al., 2009). Nevertheless, *β*-phellandrene, terpinen-4-ol and bornyl acetate were the dominant compounds in this study, and they are not major component constituents in previous reports on the EO constituent of closely related species such as *Sabina chinensis* cv. Kaizuca (Gu et al., 2018). Our study also found that all leaf samples from different harvesting times contained 26 volatile components, whereas their contents were different. Among these chemical components, the contents of *β*-phellandrene, terpinen-4-ol, *α*-elemene, and bornyl acetate in the EO from different harvest periods were quite different, of which the highest levels of *β*-phellandrene were found in May, terpinen-4-ol and *α*-elemol in March and bornyl acetate in April. The variability in chemical composition and content in aromatic plants may be attributed to differences in harvest periods, geographical factors, drying methods, and storage conditions (Zhao et al., 2021).

**Table 2:** Chemical compounds of the essential oils of *Sabina chinensis*.

No.	RT (min)	Compound name	RI	MF	Area (%)		
					March	April	May
1	5.685	Cyclofenchene	919	C_10_H_16_	0.38 ± 0.00	0.44 ± 0.02	0.49 ± 0.01
2	5.807	*α*-Thujene	924	C_10_H_16_	2.44 ± 0.22	2.64 ± 0.12	3.92 ± 0.07
3	5.961	Car-3-ene	929	C_10_H_16_	4.01 ± 0.04	2.85 ± 0.03	4.80 ± 0.05
4	6.357	Camphene	944	C_10_H_16_	0.43 ± 0.01	0.48 ± 0.00	0.53 ± 0.01
5	7.058	*β*-Phellandrene	970	C_10_H_16_	26.64 ± 0.88	33.31 ± 0.20	39.26 ± 6.67
6	7.592	*β*-Pinene	990	C_10_H_16_	4.20 ± 0.02	4.39 ± 0.01	5.69 ± 0.07
7	7.989	*α*-Phellandrene	1004	C_10_H_16_	0.88 ± 0.19	0.63 ± 0.06	0.73 ± 0.08
8	8.356	*α*-Terpinene	1015	C_10_H_16_	4.24 ± 0.50	3.35 ± 0.16	4.53 ± 3.37
9	8.736	Limonene	1027	C_10_H_16_	6.04 ± 0.32	3.77 ± 0.08	5.41 ± 2.20
10	9.373	*β*-Ocimene	1047	C_10_H_16_	0.41 ± 0.01	0.45 ± 0.00	0.40 ± 0.01
11	9.699	*γ*-Terpinene	1057	C_10_H_16_	6.07 ± 0.64	4.81 ± 0.21	6.36 ± 5.52
12	10.666	Terpinolene	1087	C_10_H_16_	2.31 ± 0.21	1.98 ± 0.06	2.48 ± 1.14
13	13.703	Terpinen-4-ol	1175	C_10_H_18_O	11.89 ± 0.05	7.57 ± 0.02	6.53 ± 1.10
14	14.256	*α*-Terpineol	1191	C_10_H_18_O	0.48 ± 0.01	0.28 ± 0.00	0.23 ± 0.00
15	16.896	Bornyl acetate	1287	C_12_H_20_O_2_	9.82 ± 0.10	10.53 ± 0.06	6.13 ± 0.09
16	19.203	Methyl (E, Z)-2,4-decadienoate	1395	C_11_H_18_O_2_	0.42 ± 0.07	0.67 ± 0.00	0.17 ± 0.02
17	21.473	Germacrene	1483	C_15_H_24_	0.36 ± 0.05	1.79 ± 0.13	0.39 ± 0.05
18	21.963	*α*-Muurolene	1501	C_15_H_24_	0.39 ± 0.01	0.73 ± 0.04	0.29 ± 0.02
19	22.398	*γ*-Cadinene	1515	C_15_H_24_	0.47 ± 0.02	0.79 ± 0.01	0.32 ± 0.01
20	22.663	*δ*-Cadinene	1523	C_15_H_24_	1.91 ± 0.04	3.97 ± 0.02	1.60 ± 0.01
21	23.468	*α*-Elemol	1549	C_15_H_24_	7.30 ± 0.57	0.83 ± 0.03	2.16 ± 1.10
22	25.454	*β*-Oplopenone	1615	C_15_H_24_	1.28 ± 0.10	1.98 ± 0.07	0.90 ± 0.03
23	26.166	*γ*-Eudesmol	1643	C_15_H_26_O	1.04 ± 0.10	0.21 ± 0.01	0.42 ± 0.03
24	26.477	Cadinol	1655	C_15_H_26_O	1.44 ± 0.16	2.60 ± 0.07	1.12 ± 0.05
25	26.763	Rosifoliol	1667	C_15_H_26_O	0.85 ± 0.08	0.37 ± 0.02	0.32 ± 0.02
26	26.847	Muurolol	1670	C_15_H_26_O	3.10 ± 0.36	4.17 ± 0.17	2.08 ± 1.13

March, April, and May show the essential oil samples from different harvest periods. Each compound of peak area is indicated as the means ± SDs with the proportions of total peak area. RT, retention time; RI, retention index; MF, molecular formula.

**Figure 4:**
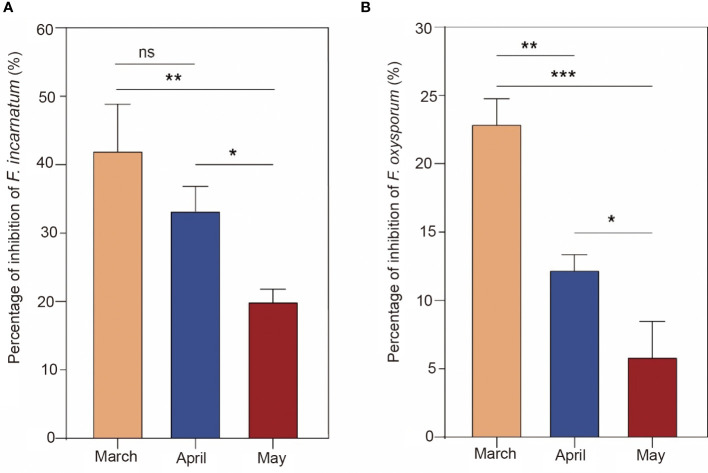
Antifungal activity of essential oils from different harvest periods (March, April, and May). The percent inhibition (means ± SDs) of *Fusarium oxysporum* **(A)** and *Fusarium incarnatum* **(B)**. The significance symbol “*” indicates a significant difference (*, *P*< 0.05; **, *P*< 0.01; ***, *P*< 0.001). The symbol "ns" indicates there was no significant difference (P> 0.05).

To further determine the difference in antifungal characteristics in different harvest periods, EO in March, April and May was tested by Petri plate assays, and the antifungal effect is shown in **Figure 4**. As a result, the inhibition rate against *F. incarnatum* by March EO treatment was higher than that by May EO treatment but was not significant by April EO treatment (*P* > 0.05). March EO treatment significantly inhibited *F*. *oxysporum* compared with April and May EO treatment (*P*< 0.05). Similarly, the treatment with the April EO sample resulted in a significantly higher inhibitory effect than the May EO sample (*P*< 0.05). Collectively, the antifungal effect of EO was different in different harvest periods, revealing that March EO had better inhibitory mycelium growth than April and May EO. This observation is in agreement with a previous study showing that *Psidium myrtoides* O. Berg EO from different harvest periods has significantly different chemical constituents and antimicrobial activities (de Macêdo et al., 2020). This dynamic variation may be related to the plant development stages and response to seasonality influenced by the accumulation and transformation of secondary metabolites (Pan et al., 2021; Dias et al., 2022).

### Correlation analysis between crucial components and antifungal properties

The major and minor compounds of EO may be related to their potential individual or synergetic antifungal effects (Aimad et al., 2022). Orthogonal partial least-squares discriminant analysis (OPLS-DA) is a supervised discriminant analysis statistical method that was employed to find potential biomarkers and reflect the metabolic variations between groups. Specifically, the OPLS-DA model was established based on 26 metabolites identified as *S*. *chinensis EO*, which had a reliable explanatory and predictive ability (*R* ^2^Y=0.973, *Q* ^2 =^ 0.937). Moreover, variable importance in projection greater than 1 (VIP>1) was calculated and considered a potential chemical marker compound (**Supplementary Table S2**). A total of 11 differential compounds were selected, including camphene (VIP=1.58), *β*-phellandrene (VIP=1.58), cyclofenchene (VIP=1.58), *α*-terpineol (VIP=1.51), terpinen-4-ol (VIP=1.51), *β*-pinene (VIP=1.46), *α*-thujene (VIP=1.45), rosifoliol (VIP=1.44), bornyl acetate (VIP=1.24), *α*-elemol (VIP=1.20), and *γ*-eudesmol (VIP=1.15). This identification of chemical markers exhibited a wide range of antimicrobial properties and applications in various commercial preparations, such as plant pathogens, food spoilage bacteria, and foodborne pathogens, which have been examined by many researchers (Haznedaroglu et al., 2001; Benali et al., 2020; Medina-Romero et al., 2022).

**Figure 5:**
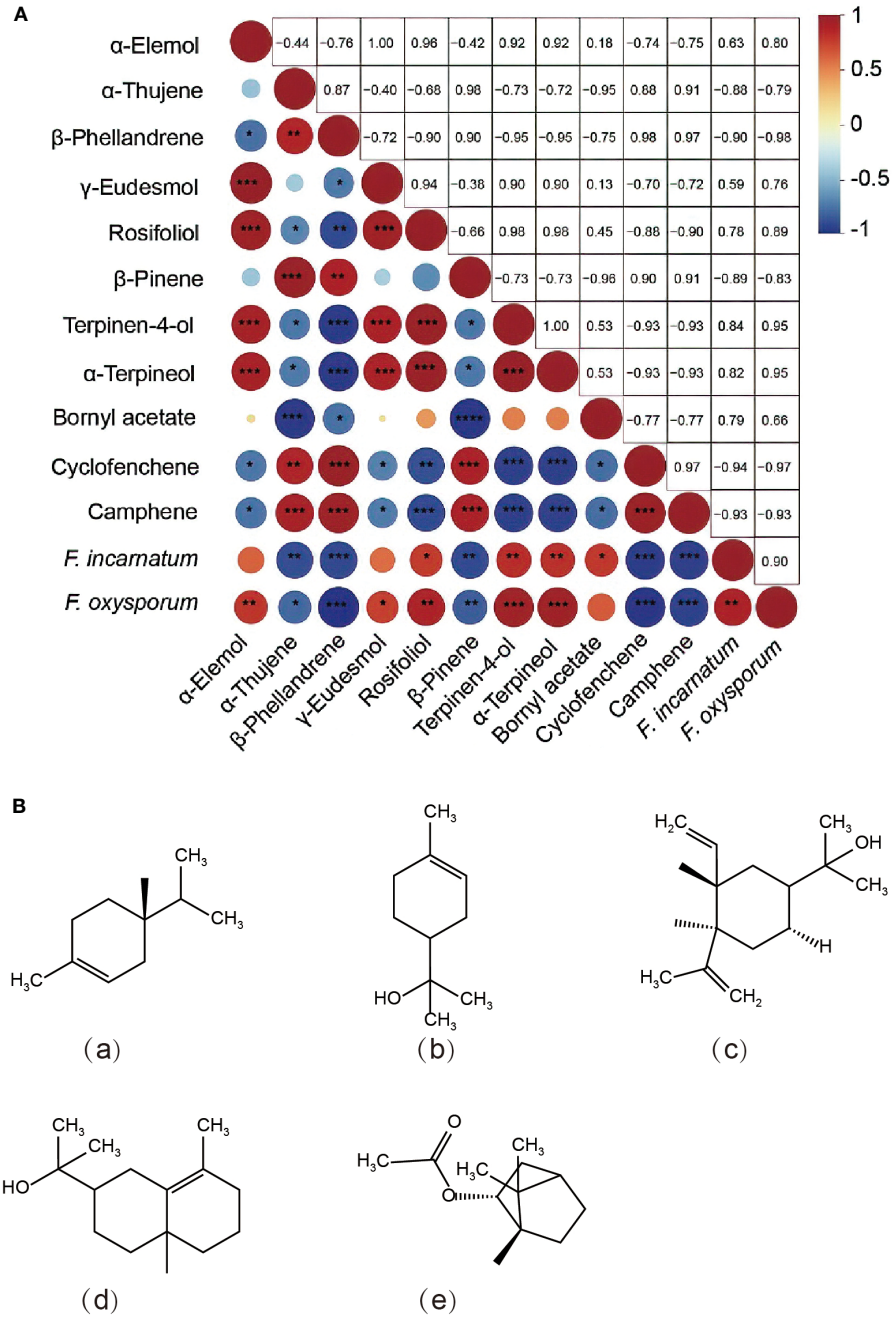
**(A)** Spearman’s correlation between variable importance in projection (VIP >1) contents and antifungal activity. Blue circles represent negative correlations, and red circles represent positive correlations. Circles with red and blue represent negative and positive correlations, respectively, and sizes represent the compound contents. **(B)** Structures of the related components found in the essential oils of *Sabina chinensis* leaves: **(a)** terpinen-4-ol; **(b)** *α*-terpineol; **(c)** *α*-elemol; **(d)** *γ*-eudesmol; **(e)** bornyl acetate. The significance symbol “*” indicates a significant difference (*, *P*< 0.05; **, *P*< 0.01; ***, *P*< 0.001).

Furthermore, a correlation analysis was performed between the mycelial growth inhibition rate and compound content by screening 11 main components in *S*. *chinensis* EO (**Figure 5**). As shown in **Figure 5A**, the contents of rosifoliol, terpinen-4-ol, and *α*-terpineol were significantly positively correlated with the antifungal level (*P*< 0.05). Among these markers, previous studies found that the *α*-terpineol and terpinen-4-ol of major components from *Artemisia* species effectively inhibited *Alternaria solani*, reaching over 60% (Huang et al., 2019). Our findings also revealed that *α*-elemol and *γ*-eudesmol were significantly positively correlated with the antifungal level of *F*. *oxysporum*, and bornyl acetate was significantly positively correlated with the antifungal level of *F*. *incarnatum* (*P*< 0.05). Previous research has also demonstrated some similar findings, with *α*-pinene, *γ*-eudesmol and bornyl acetate demonstrating antifungal activity and potential bioactivity in the control of plant diseases (Sarpeleh et al., 2009; Waikedre et al., 2012; Morales-Rabanales et al., 2021). Overall, this study speculated possible key chemical components and content and antifungal activity on enhancing antimicrobial activities and suggests that EO from *S*. *chinensis* contributed to a main source of natural plant sources (**Figure 5B**). The important next steps will be to expand the range of tested fungal species, and the formulation of a plant-derived fungicide based on *S*. *chinensis* leaf EO will be developed by screening solvents and emulsifiers and conducting toxicity tests. Then, pot experiments and field experiments will be carried out to develop future bio fungicides from plants for soil-borne pathogenic fungi control.

## Conclusion

Developing future biofungicides from the EO of plants will most likely be a critical issue for future work. In the present study, our work is the first to extend the findings of the strong antifungal activity of *S. chinensis* EO against *F*. *oxysporum* and *F*. *incarnatum* by Petri plate assays. Moreover, the chemical composition, contents and potential antifungal activity of three different harvest periods of EO were evaluated. It was of interest to note that the main components of EO, such as terpinen-4-ol and *α*-terpineol, exhibited significant antifungal activity. In summary, *S. chinensis* EO, which has been shown to have significant antifungal potential, is worth further exploration as a source of promising natural agents in soil-borne disease control and environmentally friendly agriculture.

## Data availability statement

The original contributions presented in the study are included in the article/**Supplementary Material**. Further inquiries can be directed to the corresponding authors.

## Author contributions

HF, XG, JZ, and ZZ conceived the project and designed the experiments. JZ and ZZ prepared the original draft preparation. WL and JB performed the data analyzes. YZ reviewed and edited the manuscript. HF and XG contributed to supervision, data validation, project administration, and funding acquisition. All authors contributed to the article and approved the submitted version.

## Funding

This research was supported financially by the scientific research project of the Hebei Administration of Traditional Chinese Medicine, China (Grant No. 2022098, 2022365), the Doctoral Research Foundation of Hebei University of Chinese Medicine, China (BSZ2020007, BSZ2021019), the Modern Agricultural Technology Innovation Team Project in Hebei Province, China (HBCT2018060205), the Basic Scientific Research Foundation of Hebei University of Chinese Medicine (JCYJ202206), the Traditional Chinese Medicine Resources Survey Project of China (Z135080000022), and the scientific research project of the Natural Science Foundation of Hebei Province, China (H2022423004).

## Acknowledgments

We are grateful to the teachers, administrators, and students at the Traditional Chinese Medicine Processing Technology Innovation Center of Hebei Province for their support and cooperation in this project.

## Conflict of interest

The authors declare that the research was conducted in the absence of any commercial or financial relationships that could be construed as a potential conflict of interest.

## Publisher’s note

All claims expressed in this article are solely those of the authors and do not necessarily represent those of their affiliated organizations, or those of the publisher, the editors and the reviewers. Any product that may be evaluated in this article, or claim that may be made by its manufacturer, is not guaranteed or endorsed by the publisher.
