# Supplementary material for: Essential oil from Sabina chinensis leaves: A promising green control agent against Fusarium sp
Source: Front Plant Sci. 2022 Nov 11;13:1006303. doi: 10.3389/fpls.2022.1006303 (PMC9691992; doi:10.3389/fpls.2022.1006303)
Supplement: Supplementary file 1 [file Table_1.docx]

***Supplementary Material***

**Supplementary Table 1 |** Response surface experimental design and extraction yield of essential oils from *Sabina chinensis*.

| **Run** | **Factors** | | | ***Y* (%)** |
| --- | --- | --- | --- | --- |
|  | ***A*** | ***B*** | ***C*** |  |
| 1 | 20 | 1:10 | 9 | 0.560 |
| 2 | 20 | 1:10 | 9 | 0.578 |
| 3 | 16 | 1:10 | 12 | 0.488 |
| 4 | 20 | 1:12 | 6 | 0.470 |
| 5 | 24 | 1:8 | 9 | 0.398 |
| 6 | 20 | 1:10 | 9 | 0.524 |
| 7 | 24 | 1:10 | 12 | 0.470 |
| 8 | 20 | 1:12 | 12 | 0.380 |
| 9 | 16 | 1:12 | 9 | 0.450 |
| 10 | 20 | 1:10 | 9 | 0.542 |
| 11 | 20 | 1:8 | 12 | 0.415 |
| 12 | 20 | 1:8 | 6 | 0.380 |
| 13 | 24 | 1:10 | 6 | 0.415 |
| 14 | 24 | 1:12 | 9 | 0.343 |
| 15 | 20 | 1:10 | 9 | 0.506 |
| 16 | 16 | 1:8 | 9 | 0.430 |
| 17 | 16 | 1:10 | 6 | 0.452 |

Notes: *A* is Crush degree (mesh); *B* is the Liquid/solid ratio (mL/g); *C* is the immersion time (h). *Y* is the yield of essential oils.

**Supplementary Table 2 |** Chemical markers identified for essential oils by the OPLS-DA model

| **Compound** | **molecular formula** | **VIP value** | ***P* value** |
| --- | --- | --- | --- |
| Cyclofenchene | C_10_H_16_ | 1.58 | 1.95×10^-5^ |
| *α*-Thujene | C_10_H_16_ | 1.45 | 3.34×10^-5^ |
| Car-3-ene | C_10_H_16_ | 0.63 | 6.34×10^-9^ |
| Camphene | C_10_H_16_ | 1.58 | 2.78×10^-5^ |
| *β*-Phellandrene | C_10_H_16_ | 1.58 | 1.15×10^-6^ |
| *β*-Pinene | C_10_H_16_ | 1.46 | 1.80×10^-8^ |
| *α*-Phellandrene | C_10_H_16_ | 0.66 | 1.08×10^-1^ |
| *α*-Terpinene | C_10_H_16_ | 0.33 | 1.93×10^-2^ |
| Limonene | C_10_H_16_ | 0.41 | 4.15×10^-5^ |
| *β*-Ocimene | C_10_H_16_ | 0.09 | 3.20×10^-5^ |
| *γ*-Terpinene | C_10_H_16_ | 0.25 | 1.77×10^-2^ |
| Terpinolene | C_10_H_16_ | 0.46 | 1.66×10^-2^ |
| Terpinen-4-ol | C_10_H_16_ | 1.51 | 1.61×10^-10^ |
| *α*-Terpineol | C_10_H_18_O | 1.51 | 1.49×10^-8^ |
| Bornyl acetate | C_10_H_18_O | 1.24 | 1.99×10^-9^ |
| Methyl (E, Z)-2,4-decadienoate | C_12_H_20_O_2_ | 0.77 | 1.86×10^-5^ |
| Germacrene | C_11_H_18_O_2_ | 0.03 | 1.49×10^-6^ |
| *α*-Muurolene | C_15_H_24_ | 0.33 | 2.16×10^-6^ |
| *γ*-Cadinene | C_15_H_24_ | 0.48 | 8.14×10^-8^ |
| *δ*-Cadinene | C_15_H_24_ | 0.19 | 6.40×10^-11^ |
| *α*-Elemol | C_15_H_24_ | 1.20 | 8.47×10^-7^ |
| *β*-Oplopenone | C_15_H_24_ | 0.55 | 5.35×10^-6^ |
| *γ*-Eudesmol | C_15_H_24_ | 1.15 | 5.67×10^-6^ |
| Cadinol | C_15_H_26_O | 0.33 | 5.80×10^-6^ |
| Rosifoliol | C_15_H_26_O | 1.44 | 1.56×10^-5^ |
| Muurolol | C_15_H_26_O | 0.76 | 1.38×10^-4^ |
